# Natural infection with *Trypanosoma cruzi* in bats captured in Campeche and Yucatán, México

**DOI:** 10.7705/biomedica.5450

**Published:** 2021-05-31

**Authors:** Marco Torres-Castro, Naomi Cuevas-Koh, Silvia Hernández-Betancourt, Henry Noh-Pech, Erendira Estrella, Belén Herrera-Flores, Jesús A. Panti-May, Etienne Waleckx, Javier Sosa-Escalante, Ronald Peláez-Sánchez

**Affiliations:** 1 Centro de Investigaciones Regionales "Dr. Hideyo Noguchi" Campus de Ciencias de la Salud, Universidad Autónoma de Yucatán, Mérida, México Universidad Autónoma de Yucatán Centro de Investigaciones Regionales "Dr. Hideyo Noguchi" Campus de Ciencias de la Salud Universidad Autónoma de Yucatán Mérida Mexico; 2 Facultad de Medicina Veterinaria y Zootecnia, Campus de Ciencias Biológicas y Agropecuarias, Universidad Autónoma de Yucatán, Mérida, México Universidad Autónoma de Yucatán Facultad de Medicina Veterinaria y Zootecnia Campus de Ciencias Biológicas y Agropecuarias Universidad Autónoma de Yucatán Mérida Mexico; 3 Laboratorio DYMIGEN, Mérida, México Laboratorio DYMIGEN Mérida México; 4 Grupo de Investigación en Ciencias Básicas, Escuela de Graduados, Universidad CES, Medellín, Colombia Universidad CES Grupo de Investigación en Ciencias Básicas Escuela de Graduados Universidad CES Medellín Colombia; 5 Institut de Recherche pour le Développement, UMR INTERTRYP IRD, CIRAD, Université de Montpellier, Montpellier, France Université de Montpellier 1 Université de Montpellier Montpellier France

**Keywords:** Trypanosoma cruzi, Chiroptera, infections, polymerase chain reaction, México, Trypanosoma cruzi, quirópteros, infecciones, reacción en cadena de la polimerasa, México

## Abstract

**Introduction:**

Bats have been reported as hosts of the *Trypanosoma cruzi* protozoan, the etiologic agent of American trypanosomiasis, an endemic zoonotic disease in México.

**Objective:**

To describe T. *cruzi* infection in bats from the states of Campeche and Yucatán, México.

**Materials and methods:**

Captures were made from March to November, 2017 at three sites in Yucatán and one in Campeche. Up to four mist nets on two consecutive nights were used for the capture. The bats' species were identified and euthanasia was performed to collect kidney and heart samples for total DNA extraction. *Trypanosoma cruzi* infection was detected by conventional PCR with the amplification of a fragment belonging to the T. *cruzi* DNA nuclear.

**Results:**

Eighty-six bats belonging to five families (Vespertilionidae, Noctilionidae, Mormoopidae, Phyllostomidae, and Molossidae) and 13 species *(Rhogeessa aeneus, Noctilio leporinus, Pteronotus davyi, P. parnellii, Artibeus jamaicensis, A. lituratus, A. phaeotis, Glossophaga soricina, Carollia sowelli, Chiroderma villosum, Uroderma bilobatum, Sturnira parvidens,* and *Molossus rufus)* were captured. Infection frequency by PCR was 30,2% (26/86) detected only in the renal tissue. The infected species were P. *parnellii, G. soricina, A. lituratus, A. jamaicensis, S. parvidens, C. villosum,* and *R. aeneus.*

**Conclusions:**

Our results confirmed the participation of several bat species as hosts in the T. *cruzi* transmission cycle in the region. Further studies are necessary to establish the importance of these animals in the zoonotic transmission of *T. cruzi.*

## Resumen

**Introducción.:**

Los murciélagos se han reportado como huéspedes del protozoario *Trypanosoma cruzi,* agente etiológico de la tripanosomiasis americana, enfermedad zoonótica endémica en México.

**Objetivo.:**

Describir la infección con T. *cruzi* en murciélagos capturados en los estados de Campeche y Yucatán, México.

**Materiales y métodos.:**

Se realizaron capturas de marzo a noviembre de 2017 en tres sitios de Yucatán y uno de Campeche. Para la captura se emplearon hasta cuatro redes de niebla por dos noches consecutivas. Se identificó la especie de los murciélagos capturados y se les practicó la eutanasia para recolectar muestras de riñón y corazón, utilizadas posteriormente en la extracción de ADN total. La infección con T. *cruzi* se detectó por la amplificación con PCR convencional de un fragmento perteneciente al ADN nuclear de *T. cruzi.*

**Resultados.:**

Se capturaron 86 murciélagos pertenecientes a cinco familias (Vespertilionidae, Noctilionidae, Mormoopidae, Phyllostomidae, Molossidae) y 13 especies *(Rhogeessa aeneus, Noctilio leporinus, Pteronotus davyi, P. parnellii, Artibeus jamaicensis, A. lituratus, A. phaeotis, Glossophaga soricina, Carollia sowelli, Chiroderma villosum, Uroderma bilobatum, Sturnira parvidens* y *Molossus rufus).* La PCR mostró una frecuencia de infección de 30,2 % (26/86), detectada únicamente en tejido renal. Las especies infectadas fueron P. *parnellii, G. soricina, A. lituratus, A. jamaicensis, S. parvidens, C. villosum* y *R. aeneus.*

**Conclusiones.:**

Los resultados confirmaron la participación de varias especies de murciélagos como huéspedes en el ciclo de transmisión de *T. cruzi* en la región. Es necesario realizar más estudios para determinar la importancia de estos animales en la transmisión zoonótica de *T. cruzi.*

*Trypanosoma cruzi* (of the order Kinetoplastida) is a protozoan parasite recognized as the causative agent of Chagas disease or American trypanosomiasis, zoonotic disease with relevance in areas with poverty conditions and social inequality mainly in Central American countries ^1^.

In México, approximately 1,100,000 people may be infected with T. *cruzi* and 29,500,000 are at risk of infection ^2^. The Yucatán Peninsula (in the southeast of México), which includes the states of Campeche, Yucatán, and Quintana Roo, is an area with numerous cases, mostly due to the high abundance of *Triatoma dimidiata* in the peridomestic environment and eventually inside houses ^3^. According to the Mexican *Dirección General de Epidemiología* of the *Secretaría de Salud,* in 2017 seven cases of chronic American trypanosomiasis were registered in Campeche while 86 cases of chronic American trypanosomiasis and one of the acute form were registered in Yucatán ^4^.

*Trypanosoma cruzi* infects more than 400 species of mammals ^5^. The most important natural reservoirs are armadillos, wild rodents, and opossums that help to support the wild transmission cycle ^1^^,^^6^. Other animals such as dogs ^7^, pigs, sheep, horses ^8^, and synanthropic rodents ^9^^,^^10^ have also been described as *T. cruzi* accidental hosts.

Bats are the second most diverse group (after rodents) of mammals worldwide and they are distributed in all natural areas in México ^11^. These animals have ecological and commercial relevance because they are consumers of insects known as pests and they pollinate plants used in products for human consumption ^12^. However, bats are also recognized as natural reservoirs of different viruses ^13^ and accidental hosts of bacteria ^14^ and parasites ^15^ of relevance for public and animal health.

Several studies have reported the involvement of bats in the T. *cruzi* transmission cycle, which has led to the formulation of the hypothesis that they are parasite ancestral hosts. Later, the parasite was genetically diversified and adapted to other vertebrate hosts ^16^. On the American continent, investigations with bats from Ecuador ^17^, Colombia ^18^, Argentina ^19^, México ^20^, and the United States of America ^21^ have been conducted where the importance of these mammals in the transmission risk of *T. cruzi* to other animals and human populations has been documented ^17^^-^^21^. In this context, our study aimed to describe *T. cruzi* infection in bats from Campeche and Yucatán, México, to contribute to the understanding of the intervention of bats in the *T. cruzi* transmission cycle.

## Materials and methods

### Study sites

Captures were carried out in three sites in Yucatán state and one in Campeche, México. The sites were selected for their easy access on roads close to highways and the necessary infrastructure (electricity, clean running water, and ventilated rooms) to set up a field station.

Site I (Hobonil Ranch) was located in Tzucacab municipality, Yucatán (20° 01' 00.9 " N and 89° 01' 11.8" W) and has a warm sub-humid climate with rainfall during summer and little thermal oscillation. Its average annual temperature is 26,1° C, average annual rainfall, 1,097 mm, and average elevation, 40 m above mean sea level (mamsl) 22. The ranch's vegetation is composed of medium sub-deciduous forest areas with different uses and areas with introduced species for pasture and forage ^23^.

Site II *(Ich Ha Lol Xaan* Recreational and Ecotourism Center) was located in the town of Hampolol, Campeche (19° 56' 16" N and 90° 22' 21" W). Its climate is tropical with rains in summer, an average annual temperature of 26,6° C, and an average annual rainfall of 1,088 mm. The average elevation is 10 mamsl and the vegetation is composed of several types of tropical forests (medium sub-deciduous, medium sub-evergreen, and low flood subevergreen), aquatic, and secondary vegetation ^24^.

Site III (San Francisco Ranch) was located in Panabá municipality, Yucatán (21° 21' 48.2" N and 88° 19' 23.6" W). It has a warm sub-humid climate with rains in summer, an average annual temperature of 25,6° C, and an average annual rainfall of 1,049 mm. The average elevation is 17 mamsl ^25^ and the vegetation is predominantly deciduous rainforest; however, due to agricultural and livestock activities, it has been profoundly transformed ^26^.

Site IV (Campus of Biological and Agricultural Sciences) was located in the Cuxtal Ecological Reserve, Mérida, Yucatán (20° 52' 02" N and 89° 37' 29" W). The climate is warm sub-humid with rains in summer, an average annual temperature of 26° C, and an average annual rainfall of 984,4 mm. The average elevation is 10 mamsl ^27^. The surface is mainly covered with secondary vegetation in different stages of regeneration (85 % of the total area) while the rest (15 %) is land with agricultural (cornfields, grasslands, henequenals, or other crops) or livestock uses, family gardens, streets, and houses ^28^.

### Bat captures

The captures were carried out in March (site I), May (site II), August (site III), and November (site IV) 2017 Up to four mist nets (12 m wide x 2,5 m high) were installed on each study site in two consecutive nights, situated around bodies of water, fruit trees, cave entrances, or abandoned buildings. The nets were open from 6:00 p.m. to 11:00 p.m. and checked every 20-30 min (depending on the bats activity). All the captured bats were removed from the nets and were placed in a cloth bag. Subsequently, they were transported to the field station.

### Individual variables register and biological samples conservation

At the field station, euthanasia was performed in all the studied bats following the guidelines described by the American Veterinary Medical Association ^29^. Subsequently, we identified the species of each captured individual as described by Medellín, *et al.*^30^, and Reid ^31^ and we registered their sex (male or female), reproductive status (mature or immature; in males, mature bats were individuals with descended testicles; in females, mature bats were pregnant or with alopecia areas around the nipples), and age (juvenile or adult, according to Torres-Castro, *et al.*^15^).

Heart (atria and ventricles) and kidney (cortex and pelvis) fragments from each bat were collected and deposited in a 1.5 ml microcentrifuge vial (Eppendorf™, Germany) with 99% ethanol. All the samples were stored at 4° C and transferred to the *Laboratorio de Enfermedades Emergentes y Reemergentes* at the *Centro de Investigaciones Regionales "Dr. Hideyo Noguchi"* of *Universidad Autónoma de Yucatán* where they were stored at -80° C until their use for total genomic DNA extraction.

### Total DNA extraction and quantification

Before the total DNA extraction, all tissues were washed to remove excess alcohol. Then, 25 mg of heart or kidney cut into small fragments were embedded in 25 μl of proteinase K (Omega Biotek Inc., USA) and lysis buffer; the mixture was incubated at 56° C overnight. Using a commercial kit (DNeasy Blood & Tissue Kit™, QIAGEN, Germany) and following the manufacturer's specifications. DNA extraction was performed on 86 hearts and 70 kidneys (it was not possible to collect the kidney samples from the bats from site I, Hobonil Ranch). The extracted DNA was evaluated on a spectrophotometer (NanoDrop2000™, Thermo Scientific, USA) and then stored at -79° C.

### Detection of Trypanosoma cruzi infection

The *T. cruzi* detection was performed on all collected tissues by conventional PCR using the oligonucleotides TCZ1 and TCZ2, which amplify a tandem repeated fragment of 188 base pairs (bp) belonging to a region of the nuclear DNA of T. *cruzi*^32^.

The molecular reaction included the following reagents (final concentrations): 1X PCR buffer, 2,5 mM of MgCl_2_, 0,2 μM of each oligonucleotide, 1 U Taq polymerase (Thermo Scientific, USA), 0,2 mM of dNTP's, 3 μl of template DNA (heart or kidney), and molecular biology grade water sufficient for 25 μl (final volume). The conditions in the thermal cycler were a five-minute stage at 94° C followed by 35 cycles of ten seconds at 94° C, 30 seconds at 55° C, and 30 seconds at 72° C. The final extension was for five minutes at 72° C.

All reactions included positive (genomic DNA extracted from rodent organs experimentally infected with a T. *cruzi* lineage I) and negative controls (all reaction reagents but with no template DNA). Electrophoresis was performed on 1% agarose gels stained with ethidium bromide. The gels were then visualized in a photo-documentation system (Bio-Rad, USA) to record the results.

### Statistical analysis

We used descriptive statistics to determine the T. *cruzi* infection frequency and the frequency of each variable (age, sex, and reproductive condition) collected in the studied bats. Additionally, we explored the association strength of each variable with the infection frequency using a chi-square test (X^2^). Data were analyzed on the Epiinfo™ program (version 7.2.3.0) (CDC, USA); p<0,05 was considered the value of statistical significance.

### Bioethical guidelines

The Mexican *Secretaria de Medio Ambiente y Recursos Naturales* approved the extraction of the captured animals (minutes SGPA/DGVS/ 03705/17 and SGPA/DGVS/01186/17). The Bioethics Committee of the *Facultad de Medicina Veterinaria y Zootecnia* (minutes CB-CCBA-I-2018-001) approved the capture, sacrifice, and biological sampling of the studied bats.

## Results

Eighty-six bats of 13 different species belonging to five families were captured. Table 1 shows the family with the greatest richness: Phyllostomidae with eight distinct species and the species with the highest number of individuals captured: A. *jamaicensis,* present in all study sites.

**Table:** 1. Family, species, total number of captured and infected bats per study site in Yucatán and Campeche, Mexico

Family	Species	Site I	Site II	Site III	Site IV	N	Infected by species (n) (%)
		n	n	n	n		
Vespertilionidae	*Rhogeessa aeneus*	0	0	1	0	1	1 (100)
Noctilionidae	*Noctilio leporinus*	0	6	0	0	6	0 (0)
Mormoopidae	*Pteronotus davyi*	2	0	0	0	2	0 (0)
	*Pteronotus parnellii*	0	6	0	0	6	4 (66.7)
Phyllostomidae	*Artibeus jamaicensis*	6	7	12	20	45	10 (22.2)
	*Artibeus lituratus*	0	1	1	0	2	2 (100)
	*Artibeus phaeotis*	3	0	0	0	3	0 (0)
	*Glossophaga soricina*	0	1	3	1	5	4 (80)
	*Carollia sowelli*	0	2	0	0	2	0 (0)
	*Chiroderma villosum*	0	1	6	0	7	4 (57.1)
	*Uroderma bilobatum*	0	1	0	0	1	0 (0)
	*Sturnira parvidens*	0	1	0	0	1	1 (100)
Molossidae	*Molossus rufus*	5	0	0	0	5	0 (0)
Total		16	26	23	21	86	26

PCR results showed an overall infection rate of 30.2% (26/86). All positive reactions corresponded to renal tissue extractions. The species with infected individuals were: A. *jamaicensis, G. soricina, C. villosum, P. parnellii, A. lituratus, R. aeneus,* and *S. parvidens,* each one with different infection frequencies according to the number of captured individuals for each species (table 1). Of the infected bats, 16 were captured at site III (61.5%) and 10 at site II (38.5%).

Table 2 shows the values and frequencies of the individual variables considered in the bat population under study, as well as those from bats infected with T. *cruzi.* The %^2^ test showed no significance for any of the evaluated cases (p>0.05).

**Table 2:** Values and frequencies of the bats captured and infected with *Trypanosoma cruzi* in sites of Yucatán and Campeche, México

	Captured bats* n %	Bats infected with Trypanosoma cruzi** n %
Study sites		
I	16 (18.6)	0 (0)
II	26 (30.2)	10 (38.5)
III	23 (26.8)	16 (61.5)
IV	21 (24.4)	0 (0)
Sex		
Male	48 (55.8)	10 (38.5)
Female	38 (44.2)	16 (61.5)
Age		
Juvenile	28 (32.6)	8 (30.8)
Adults	58 (67.4)	18 (69.2)
Reproductive condition		
Active	50	12 (46.2)
Inactive	36	14 (53.8)

* n=86

** n=26

## Discussion

We captured individuals from five families and 13 species of bats, i.e., 20.3% of the total number of species found in the Yucatán Peninsula ^33^. The most abundant species by capture was *A. jamaicensis,* which is widely distributed in the Yucatán Peninsula as it is tolerant to ecosystem fragmentation and can colonize a wide variety of natural or artificial shelters ^34^.

In México, there are reports of *T. cruzi* natural infection in several bat species ^20^^,^^35^^,^^36^. In our study, five species were found to be infected in each state (Campeche and Yucatán) amounting to a total of seven distinct infected species in all the study sites. This diversity of infected species captured at specific sites underlines the need to evaluate T. *cruzi* distribution in the different epidemiological environments that could be influencing the transmission dynamics in bat populations, such as the abundance of other vertebrate hosts and the circulating species of insect vectors, as well as bat behavior and ecology ^20^^,^^37^^,^^38^.

In a first study conducted with bats from Molas, Yucatán, infection with T. *cruzi* was described in *A. jamaicensis, A. lituratus,* and *S. parvidens*^35^. In this sense, our reports for C. *villosum, G. soricina,* and R. *aeneus* are the first in Yucatán. Additionally, in another study in bats from Calakmul, Campeche, *A. jamaicensis, A. lituratus, S. parvidens, S. ludovici, C. brevicauda,* and *Myotis keaysi* were found to be infected ^20^ while here we present unprecedented reports of *T. cruzi* infection in *P. parnellii* and *G. soricina* individuals captured in Campeche.

In a study on bats from Morelos (central México), *T. cruzi* infection was described in *A. jamaicensis, G. soricina, S. parvidens,* and *Choeronycteris Mexicana*^36^. In the context of these findings and the results from studies on bats in Yucatán ^35^ and Campeche ^20^, we present here the first evidence of *T. cruzi* infection in *C. villosum* individuals captured in Mexico. Previously, *Pteronotus parnellii* had been reported as parasitized by *T. cruzi* in the state of Morelos ^36^.

At the international level, there are also reports of *T. cruzi* infection in bats of different genera. For example, in Colombia, infected individuals of *Carollia, Desmodus, Glossophaga, Noctilio, Peropteryx, Phyllostomus,* and *Artibeus* genera have been reported and *A. lituratus* and *C. villosum* species were suggested as *T. cruzi* accidental hosts ^18^, which coincides with our results. Likewise, in Ecuador, infections in individuals of *Artibeus* and *Myotis* genera and G. *soricina* species have been reported ^17^; this last species was also detected as infected in our study. In Brazil, *A. lituratus* and *G. soricina* were registered as accidental hosts for T. *cruzi*^39^. In South America as well, specifically in Perú, infections in *Phyllostomus, Diaemus, Trachops,* and *Desmodus* specimens have been reported ^37^. However, none of these infected species coincide with those found in the bat population studied in Campeche and Yucatán, although it is important to note that not all species circulate in both study regions (Perú and México).

There are several hypotheses about *T. cruzi* transmission routes to bat populations. These mammals are usually established in caves, trees, or artificial constructions (buildings and houses), places where *T. dimidiata* and other triatomines, as those reported in Ecuador, such as *Cavernicola pilosa* (associated with *Myotis* sp.) and *Triatoma dispar* (associated with *Molossus molossus)* have been detected ^17^. *Cavernicola pilosa* has also been found in roost sites of at least nine bat species within five families ^40^^,^^41^.

These findings are relevant for human health because triatomines associated with bats species might opportunistically feed on and transmit trypanosomes to humans ^17^^,^^38^ and they also demonstrate that bats and triatomines share the same area and shelters considered to be restricted to sylvatic environments ^17^; therefore, there is a probability of T. *cruzi* vectorial transmission to bat populations ^42^^,^^43^.

In this context, in a study in which massive sequencing (12S RNA gene) was used to analyze the abdominal contents of 14 triatomines *(T. dimidiata)* collected in Yucatán, at least 14 species of vertebrate animals were described as food sources for these insects, among them, *Artibeus* bats; consequently, the authors concluded there is the probability that this bat genus participates in the stability of the *T. cruzi* transmission cycle contributing to the parasite enzootic expansion in the wild environments of the study region ^5^^,^^16^^,^^44^ since some *Artibeus* species (including A. *jamaicensis)* have been captured around and inside rural houses in the Yucatán Peninsula ^35^.

Infections in bat species belonging to the insectivorous trophic level may have been caused by the intake of triatomines hosting the parasite. Thomas, *et al.*^45^, confirmed this in *Artibeus, Carollia, Glossophaga,* and *Molossus* individuals when triatomines experimentally infected with T. *cruzi* were given to them for consumption. Finally, T. *cruzi* transmission during the gestation period (vertical or congenital), or the lactation stage, has also been described in bats ^46^.

The presence of T. *cruzi* DNA was only detected in the renal tissue of the studied bats. Although unexpected, as T. *cruzi* usually invades the cardiac cells ^1^, this may be explained by many factors, among them, the virulence of infectious strain ^19^. In this context, in experimental conditions is has been shown that a limited distribution in the affected vertebrate hosts' organism can be generated depending on this characteristic ^19^^,^^47^^,^^48^. Likewise, T. *cruzi* did not persist in the cardiac tissue of experimentally infected mice ^49^^,^^50^.

Further studies are necessary to determine the infective strains in the bat populations of the region, as well as the distribution and the damage that the parasite may cause in organs and tissues ^16^. Infected bats with T. *cruzi* may have implications for public health ^5^^,^^16^^,^^21^^,^^37^ considering that T. *dimidiata* lodges in cracks or dark spaces in rural homes to feed on its inhabitants ^42^^,^^43^. In this respect, Ramírez, *et al.*^38^ have shown through cloning and blood culture the infection with T. *cruzi* strains transmitted from bats to humans. Additionally, Villena, *et al.*^37^, suggest that bats with chronic infections in their salivary glands can contaminate fruits and vegetables with viable parasites deposited in their saliva, which allows T. *cruzi* transmission to human populations and other susceptible animals through the intake of contaminated food.
